# Beyond the numbers: the importance of contextual data when reusing blood pressure data from electronic health records

**DOI:** 10.3389/fdgth.2025.1664213

**Published:** 2025-09-03

**Authors:** Marieke E. Grünewald, Erik Koomen, Lex M. van Loon, Ankit Gupta, Robin W. M. Vernooij, Wouter W. van Solinge, Teus Kappen, Saskia Haitjema

**Affiliations:** ^1^ Central Diagnostic Laboratory, University Medical Center Utrecht, Utrecht University, Utrecht, Netherlands; ^2^ Pediatric Intensive Care Unit, Wilhelmina’s Children’s hospital, University Medical Center Utrecht, Utrecht University, Utrecht, Netherlands; ^3^ Intensive Care Center, University Medical Center Utrecht, Utrecht, Netherlands; ^4^ Cardiovascular and Respiratory Physiology, Technical Medical Centre, University of Twente, Enschede, Netherlands; ^5^ Siemens Healthcare Private Limited, Bangalore, India; ^6^ Department of Nephrology, University Medical Center Utrecht, Utrecht University, Utrecht, Netherlands; ^7^ Department of Anesthesiology, University Medical Center Utrecht, Utrecht University, Utrecht, Netherlands; ^8^ Directorate Information Technology, University Medical Center Utrecht, Utrecht University, Utrecht, Netherlands

**Keywords:** electronic health records, data reuse, EHDS, semantic interoperability, blood pressure, fit for purpose, contextual data

## Abstract

With the digitization of health records, the reuse of Electronic Health Record (EHR) data has become increasingly prevalent in research. Using blood pressure as a case study, this paper examines the complexities and practical realities of reusing EHR data, emphasizing the importance of contextual information for accurate interpretation. Although blood pressure data derived from EHR systems may appear straightforward—often captured by machines or derived from standardized workflows—their reuse is frequently complicated by variability in measurement methods and clinical contexts, which can produce seemingly similar but clinically distinct blood pressure readings. The paper begins with the physiology of blood pressure and the various techniques used to measure it. This is followed by an analysis of different clinical settings—i.e., the different pathophysiological situations—that may affect both measurement practices and data interpretation. The paper then explores how these measurements are recorded in EHR systems and concludes with practical guidance to support researchers in identifying blood pressure data that are truly fit for the intended research purpose. By acknowledging the inherent complexities of healthcare data and making informed data selection decisions, researchers can better harness the potential of EHRs to generate meaningful insights that ultimately improve patient care.

## 1 Introduction

With the digitization of health records, an increasing number of databases containing valuable healthcare data have become available for (re)use in research beyond the original intent for which the data was collected (1). The future European Health Data Space (EHDS) will accelerate health data exchange across Europe, enabling researchers to gain access to much more, and more diverse, health data (2). This presents new opportunities: i.e., uncovering patterns and correlations that may lead to new treatment plans, improving patient care, and supporting more effective health policies (1).

Electronic Health Record (EHR) data are typically generated in clinical processes (e.g., diagnosis making, therapeutic decisions). Besides qualitative data such as clinical notes, EHR data may include a variety of quantitative measurements: from laboratory results such as hemoglobin values to vital signs such as blood pressure. These measurements may appear straightforward and ideal for (re)analysis as they are often captured by machines or derived from protocolized workflows. Unfortunately, such EHR data are far more complex than they appear, which complicates their immediate reusability (3). A single type of measurement can have multiple methods to acquire its values and can often be used for different clinical purposes. This makes the interpretation of these values highly sensitive to the clinical context in which the measurements were made. A lack of understanding of these contextual factors makes it impossible to first correctly understand the data within the clinical context; and next to be able to select which data are relevant and appropriate to answer one's research question.

This paper aims to illustrate the complexity of health data reuse and the practical realities of working with real-world clinical data, and that careful interpretation and evaluation is necessary to create value from EHR data. Although the considerations in this paper are important for almost all data coming from measurements performed in medical settings, we will focus on blood pressure measurements as an example.

We will first discuss the physiology of blood pressure, followed by how blood pressure can be measured. Then we distinguish the different clinical settings—i.e., the different pathophysiological situations—that may affect both measurement methods and interpretation. We will then discuss how measurements end up in EHR databases and conclude with concrete examples and practical guidance to support researchers in selecting blood pressure data that are truly fit for answering their research questions.

## 2 The basic physiology of blood pressure

The heart pumps blood through the blood vessels of the entire circulatory system, delivering oxygen and nutrients to all the organs and tissues, and, at the same time, evacuating waste products. The force exerted by the circulating blood on the blood vessels walls is known as blood pressure. In clinical practice, blood pressures are used as an indicator of blood vessel wall stress, which may result in damage. In addition, blood pressure is used as a surrogate of blood flow within the circulatory system, as blood pressure, unlike blood flow, can easily be measured *in vivo*.

During each cardiac cycle, blood flows through the arteries in a pulsatile fashion. Systolic pressure represents the peak pressure when the left ventricle of the heart contracts and ejects blood into the aorta. Diastolic pressure reflects the lowest pressure during cardiac relaxation, when the ventricles refill. The difference between these two values is called the pulse pressure.

Blood pressure exhibits variability patterns that reflect the body's dynamic efforts to preserve homeostasis through continuous autoregulation (4). A short-term regulatory system (seconds to minutes) can be distinguished from a long-term regulatory system (days to weeks) (5).

Furthermore, blood pressure changes with age: systolic blood pressure rises between the ages of 30 and 84 years whereas diastolic blood pressure increases until the fifth decade of life, then slowly decreases after the age of 60 (6). Moreover, the blood pressure trajectories of men and women differ notably. As early as the third decade of life, women exhibit faster rates of blood pressure elevation compared to men (7). Women generally have lower blood pressure than men until menopause, after which their systolic blood pressure surpasses that of men (8).

## 3 Measuring blood pressure

### 3.1 The development of blood pressure measurement

The mercury sphygmomanometer has been the traditional method for blood pressure measurement (9). Introduced by Scipione Riva-Rocci in 1896, it includes compressing the brachial artery non-invasively with a cuff until blood flow stops, then gradually releasing the pressure and detecting the return of flow with the fingertips, providing a systolic pressure reading. The sphygmomanometer included a column of mercury to display the pressure (10), which is why blood pressure is denoted in millimeter mercury rather than Pascal, the SI unit of pressure [even though mercury sphygmomanometers are not used anymore (4) due to safety and economic concerns about the effects of mercury (11)]. Riva-Rocci's name can still be found in the frequently used abbreviation RR to denote blood pressure in patient charts (e.g., RR 120/80 mmHg).

In 1905, Nikolai Korotkoff discovered that listening to the brachial artery using a stethoscope during cuff deflation revealed more detailed information. Specifically, he identified a series of sounds, now known as Korotkoff sounds, that occur as blood begins to flow again during deflation. In the manual auscultation method, the first appearance of these sounds indicates the point where the pressure from each heartbeat is strong enough to overcome the cuff's compression, corresponding to systolic blood pressure. As the cuff further deflates, these sounds disappear, corresponding to diastolic pressure, as the cuff pressure is no longer strong enough to compress the artery when there is no force from the heartbeat (12). Blood pressure is most of the time expressed as systolic over diastolic blood pressure (SBP/DBP). Over time, mercury manual auscultatory devices have been replaced by aneroid auscultatory devices, which require regular calibration. The accuracy of these devices is highly dependent on operator skill, particularly in detecting Korotkoff sounds. This innovation remains the foundation of modern blood pressure measurement.

### 3.2 Modern blood pressure measurement

Manual auscultatory blood pressure measurement is now being replaced by automatic blood pressure measurement devices that follow the same basic principles as manual methods, particularly in the usage of a cuff and inflation to temporarily halt the blood flow in the branchial artery (9). However, rather than relying on a stethoscope to detect Korotkoff sounds, these devices use electronic sensors to analyze oscillations in cuff pressure during deflation (13). These oscillations form an envelope used by algorithms to estimate systolic, diastolic, and mean pressures. The algorithms are trained on limited datasets and often validated either against manual or invasive BP measurements (only recently). Increasingly, manufacturers validate their algorithms using invasive standards to reduce discrepancies between non-invasive and intra-arterial BP readings. However, differences in algorithm design persist, especially across age groups (neonates, pediatrics, adults). The auscultatory method, which involves using a stethoscope to listen to Korotkoff sounds, is still preferred in certain situations, such as when measuring blood pressure in patients with atrial fibrillation (4) and is still seen as the reference standard in the treatment of hypertension.

Invasive blood pressure measurement can provide continuous, real-time blood pressure monitoring by directly measuring the pressure inside an artery. Invasive blood pressure measurement is considered the reference standard in critical care. A cannula is inserted into an artery, typically the radial artery (femoral or brachial arteries can also be used), and is connected to tubing filled with heparinized saline. The fluctuations in blood pressure cause pulsations in the saline column and displacement of a diaphragm which has an in-built gauge (“Wheatstone Bridge”). The monitor amplifies the signal from the transducer, filters out noise, and generates a pressure waveform. This waveform can be displayed in real time, along with the digital values for systolic, diastolic, and mean arterial pressure (14). For accuracy, transducers must be zeroed at the level of interest, typically the heart level, and the lines must be fluid-filled and air-free. Improper leveling or air in the system can dampen waveforms and yield inaccurate values. Regular calibration (at least every 24 h) is required. These lines are also used for arterial blood sampling. Newer methods of continuous, non-invasive blood pressure management are available, but typically not yet applied at a large scale. An overview of blood pressure methods is provided in Table 1.

**Table 1:** An overview of blood pressure methods.

Method	Access route	Measurement Principle	Frequency	Examples/Devices	Remarks
Auscultatory (Korotkoff) (AU)	Non-invasive	Occlusion (sound)	Intermittent	Manual cuff with stethoscope	Reference method, requires operator skill
Oscillometric (OSC)	Non-invasive	Occlusion (oscillations)	Intermittent^a^	Automatic monitors, ABPM, home devices	Most commonly used; less accurate in arrhythmia or vascular pathology
Volume clamp (finger cuff) (VC)	Non-invasive	Volume regulation	Continuous	Finapres, CNAP	Suitable for continuous monitoring; fairly accurate but limited availability
Pulse Transit Time (PTT)	Non-invasive	Pulse propagation (timing)	Continuous	Wearables, R&D setups	Not standard in clinical care; estimation only, requires calibration
Arterial catheter (A-line) (INV)	Invasive	Direct pressure measurement	Continuous	ICU, OR, emergency settings	Highly accurate; risk of complications; used in specialist care

^a^ Oscillatory measurements can be performed in a “continuous mode” where intermittent measurement are automatically performed at regular intervals, typically between 1 and 60 min.

R&D, research & development; ICU, intensive care unit; OR, operating room; ABPM, ambulatory blood pressure monitoring; CNAP, continuous noninvasive arterial pressure.

### 3.3 Measurement considerations

A valid blood pressure reading accurately represents the patient's cardiovascular status and complies with regulatory and methodological standards. Differences have been observed between invasive and non-invasive blood pressure measurements: invasive blood pressure measurement is generally considered more accurate than non-invasive methods. Non-invasive systolic values tend to be overestimated, while diastolic and mean arterial pressure (MAP) readings are often underestimated. Nevertheless, non-invasive methods generally show good correlation with invasive readings across time points, supporting their continued use in clinical practice (15). There are ISO standards for quality assurance of blood pressure devices (e.g., ISO 81060-1 through 7). However, multiple factors can influence the accuracy or interpretation of a blood pressure reading, as detailed in Supplementary Table S1.

In clinical care, blood pressure measurements are particularly used for screening for a condition (first measurement), or diagnosis or monitoring of one (using follow-up measurements). Age-specific reference ranges are available for systolic and diastolic blood pressure, that may indicate hypotension (low blood pressure), normotension (normal blood pressure), or hypertension (high blood pressure).

### 3.4 Screening for a condition

Blood pressure is frequently used as a simple and quick screening tool for general health status, both in general practice, outpatient clinics, on clinical wards, and in emergency healthcare settings. Low blood pressure can manifest as a feeling of malaise, along with other symptoms like dizziness, lightheadedness and fatigue, and high blood pressure may present with headaches, all frequently occurring complaints at GPs and outpatient clinics. Blood pressure for screening purposes is typically performed once, using an oscillometric measurement. Accurate interpretation of this measurement requires considering the patient's age, gender, and overall clinical situation, along with clinical expertise to determine what is normal for that individual. Since the normal blood pressure reference range is relatively broad, a reading may still fall within this range but be significantly lower or higher than the patient's usual levels, making it a cause for concern. Moreover, there may be a suspicion that the patient's situation may change, which calls for repeating the blood pressure measurement.

### 3.5 Diagnosis of a condition

If blood pressure is high, a follow-up measurement may be taken to diagnose a disease called “essential hypertension”, which means that no underlying cause for the hypertension could be detected. With an estimated 1.28 billion adults affected worldwide—nearly half of whom are unaware of their condition (16)—an early diagnosis of hypertension is essential for effective management (17). Hypertension progresses without noticeable symptoms while causing progressive damage to blood vessels over time and thus increases the risk of cardiovascular diseases, including life-threatening conditions such as heart attacks, strokes, and kidney damage.

In the hypertension clinic, blood pressure is typically measured according to specific guidelines (18). First, consecutive measurements, such as 24 h blood pressure measurement, where patients wear a portable device that measures an automated blood pressure every 15 min for 24 h, or a 30 min measurement in continuous mode, where automated blood pressure is measured every 3 min for 30 min, may be needed to definitively make the diagnosis.

When diagnosing hypertension, the focus is on whether blood pressure exceeds established thresholds. Conditions such as stress, pain, fever, or pharmacological interventions can cause transient blood pressure elevations. Differentiating temporary from chronic changes is vital for diagnosis and treatment. The threshold is dependent on the measurement method; office, home, and ambulatory blood pressure measurement threshold differ (Table 2).

**Table 2:** Comparison of office, home, and ambulatory blood pressure measurements thresholds for elevated blood pressure and hypertension by the ESC (18).

	Office BP (mmHg)	Home BP (mmHg)	Daytime ABPM (mmHg)	24 h ABPM (mmHg)	Night-time ABPM (mmHg)
Reference					
Non-elevated BP	<120/70	<120/70	<120/70	<115/65	<110/60
Elevated BP	120/70-<140/90	120/70-<135/85	120/70-<135/85	115/65-<130/80	110/60-<120/70
Hypertension	≥140/90	≥135/85	≥135/85	≥130/80	≥120/70

ABPM, ambulatory blood pressure monitoring; BP, blood pressure.

There is an ongoing discussion about whether women should have lower diagnostic thresholds than men, as they may face health risks at comparatively lower blood pressure levels (19).

As monitoring over time is needed, accurate measurements are key to drive pharmaceutical treatment with blood pressure lowering medication. Manual measurements are preferred because of their accuracy, but out-of-office measurements, such as home and ambulatory blood pressure monitoring, are increasingly recommended, as they reduce the risk of misdiagnosis due to phenomena like white-coat or masked hypertension (18, 20) (Supplementary Table S1).

### 3.6 Monitoring of a condition

A follow-up measurement may also be taken to either monitor while treating a disease-causing hypotension or hypertension, or during an induced condition such as anesthesia. In critical care and surgical environments, blood pressure measurements are vital for monitoring patient stability.

Acute hypertension may have several origins and may lead to acute *vessel damage*, such as brain bleeds if left untreated. While titrating blood pressure downwards, the culprit of the hypertension needs to be treated to prevent the damage. On the other hand, hypotension may be associated with acute illness as it causes inadequate *tissue perfusion*, which may lead to deprived delivery of oxygen and nutrients: shock. Shock can have different origins, but, too, will not resolve by restoring blood pressure unless the underlying culprit is treated (21). Blood pressure is measured frequently in these situations, up to every few minutes. In severe cases, invasive methods can be used, e.g., on the intensive care unit (ICU).

In the intensive care unit, continuous blood pressure monitoring is especially important for critically ill patients, whose blood pressure often fluctuates due to their unstable condition. For these patients, goal-directed therapy guided by continuous blood pressure monitoring using invasive measurements is essential to ensure effective circulatory management and to make timely interventions aimed at stabilizing their condition (22).

Anesthesiologists measure blood pressure using an automated device before surgery to establish a baseline, which is then used for comparison during the procedure. Throughout surgery, blood pressure is continuously monitored to maintain hemodynamic stability, with adjustments made as necessary to address any fluctuations. Invasive methods, such as arterial lines, provide real-time blood pressure data during surgery, allowing for precise regulation. After the operation, the anesthesiologist continues to monitor blood pressure during the recovery period to ensure stability. Maintaining appropriate blood pressure levels is crucial for optimal recovery and preventing complications.

**Figure 1:**
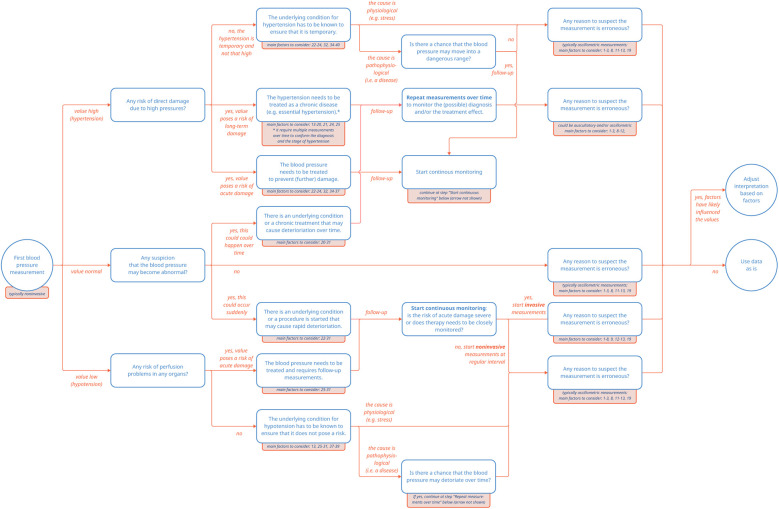
Pathways of blood pressure measurements. The numbered factors to consider correspond to the entries listed in Supplementary Table S1.

In summary, both hypotension and hypertension may warrant treatment, yet for two entirely different reasons. In the case of hypotension, the underlying condition causing the low blood pressure is treated to prevent damage to the hypoperfused organs. On the other hand, in the case of hypertension, either acute or chronic damage to the vessels itself is at stake and hypertension may be diagnosed and treated as a condition on its own. These different reasons for measuring blood pressure highlight how different clinical scenarios may generate seemingly similar blood pressure data points in clinical care (Figure 1).

### 3.7 Capture and storage of blood pressure measurements in EHR systems

Most EHR systems capture and store blood pressure measurements along with the corresponding metadata in a measurements table. This table may include also other measurements like length, weight and risk scores like Glasgow Coma Scale, so filtering on measurement type may be needed. Blood pressure data is typically stored as systolic (SBP) and diastolic (DBP) values in separate numeric fields or sometimes as a combined character string (e.g., “120/80”). Most health devices will adhere to the international communication standard (i.e., ISO/IEEE 11073). Regardless of the entry method, blood pressure values are typically recorded as whole numbers, without the use of decimals to indicate measurement precision. When unit of measurement is not explicitly stated, is it generally safe to assume it to be millimeters of mercury (mmHg). Data interoperability models like FHIR, OMOP, and openEHR support the capture of metadata; however, these fields are often left unfilled in practice, as structured data entry can be time-consuming and its adoption varies widely among clinicians (23). Measurement tables in EHR databases may therefore contain more or less of the following metadata elements per observation: a measurement identifier, identifier of the person or device that captured the measurement (“performer” or “device”), measurement date and time, and a measurement method.

According to local IT infrastructure and availability of devices that are interoperable with the EHR system, both people and devices may record readings into the EHR. Auto-recorded values from devices may or may not be manually verified by people, according to local protocol. In addition, the specific device used for measurement is rarely documented, limiting traceability and posing challenges for retrospective quality control if equipment malfunction or miscalibration is discovered, introducing the risk of systematic bias in downstream analyses. The method of measurement, whether invasive or non-invasive, may be specified. In case of a manual recording, the identity (code) of the healthcare provider associated with the measurement is often recorded; however, this typically refers to the individual or machine who entered the data into the database, which may not necessarily be the person or machine who performed the measurement. For manually entered data, the recorded timestamp, too, frequently reflects the time of data entry rather than the actual time of measurement, especially when entries are made in batches following clinical rounds (24).

The increasing use of consumer-grade wearables and cuffless blood pressure adds complexity. While non-invasive techniques have advanced considerably, limitations in calibration and traceability hinder their clinical applicability. Cuffless monitoring—often based on optical or tonometric data—still lacks the precision and regulatory backing necessary for use as a primary diagnostic tool. As such, while promising, these technologies are not yet a replacement for validated clinical methods. However, IT solutions, both in-hospital, transmural and patient-oriented (such as CE or non-CE marked consumer monitoring apps), may facilitate capturing automated or manual capture and storage of patient-derived blood pressure data. Different healthcare professionals, departments and hospitals may deploy different protocols for how and when to incorporate these data into their clinical decision making.

As the previous paragraph exemplifies, clinical practice is evolving. This includes changing guidelines and practices that may further influence and shape the clinical context in which blood pressure measurements are captured. For example, in cardiovascular risk management, recent changes in clinical guidelines reflect an evolving understanding of cardiovascular risk. For example, the 2017 ACC/AHA guidelines lowered the threshold for hypertension diagnosis from 140/90 mmHg to 130/80 mmHg. Implementation of these new guidelines and the accompanying efforts towards target attainment may have lowered both the mean and the median blood pressure in preventive care in recent years, thereby for example affecting frequently used imputation methods of missing data.

In summary, even when interoperability standards are sufficiently deployed, the accompanying recorded metadata may be insufficient for reconstructing full measurement conditions that are needed to facilitate reuse of data. Below, we will further dive in to provide some practical examples on how to select fit-for-purpose data based on contextual clues.

## 4 Preparing the ideal blood pressure data for reuse

For researchers reusing EHR data, specifically (data) scientists working on big datasets, blood pressure data is likely presented in long-format tables, with values linked to distinct patient measurements across large populations. However, in a data table, these values are disconnected from the clinical context they were measured in. Unlike clinicians, scientists that reuse these data do not see the individual patient, hear their symptoms, or have access to the description of a clinical context and the clinical rationale for which the data was captured. Assuming all data points are equivalent in meaning or quality risks misinterpretation. By now, the reader knows a blood pressure measurement that was recorded in an emergency department during a crisis is not the same as one taken during a calm check-up visit, even if the data and metadata appear identical and technically sound in the dataset. Without awareness of the meaning of the variables, what information is missing, and how that may affect interpretation, an analysis can be technically correct but lack clinical meaning.

First, a clearly defined research question is essential. It not only shapes the analytical approach but also guides which data should be used. This way, data selection is not treated as a fixed starting point, but rather as a design choice aligned with the study's purpose. This principle, referred to as “fit-for-purpose”, emphasizes that data must be both relevant and appropriate for the question being addressed (25). Furthermore, fit-for-purpose acknowledges that some research questions may tolerate more variability in measurement or documentation than others. Exploratory studies, for instance, may be less sensitive to occasional inconsistencies, while evaluations of treatment effects or safety may require highly standardized and reliable data. Meaningful analysis depends on data that are not only of high quality but also contextually aligned with the research question; without this contextual alignment, even the best data may fail to produce meaningful results (26). Fortunately, on top of metadata, contextual clues may exist in the EHR system to help researchers assess this alignment.

A key component of the fit-for-purpose principle on a dataset level is considering the source of the data. While issues such as selection bias and limited generalizability are common to all database research, they deserve particular attention when EHR data are used for secondary purposes. Using data that is primary collected for research, the decision to collect data using specific devices or describe specific metadata can be made before data collection, leading to generally a more harmonized and complete dataset for the specific research question. Unlike primary data collection, EHR data is not designed for research purposes, and its structure often reflects e.g., clinical workflows rather than study design. For instance, including only patients with complete longitudinal records can unintentionally overrepresent sicker individuals, who typically have more frequent and detailed documentation (27). Similarly, data obtained from specific settings, such as long-term care facilities or specialized clinics, may not be representative of broader patient populations or be generalizable to the wider population due to differences in clinical practices and patient demographics. Understanding the background and structure of the contributing health organization is essential to determine whether it can provide the target cohort and relevant clinical data (28).

On an individual datapoint level, contextual clues may be found in different areas. First, the frequency of the measurements itself may point towards a clinical situation: if 10 measurements are available in a 30 min timeframe, these are probably part of an automated measurement in “continuous mode”. If blood pressure measurements have a high frequency of for example one every minute, they were probably measured invasively, which points towards a monitoring situation. Second, some contextual information can be inferred from associated data in the EHR system. For example, the performer of the measurement could be linked to the medical discipline or department. Moreover, blood pressure measurements can be linked to encounters, further specifying if the measurement occurred during an inpatient stay, outpatient visit, or emergency episode. In addition, linkage can be made to medications, which may indicate the use of antihypertensive drugs during the measurement.

### 4.1 Example use cases

A 30-year-old woman in her third trimester visits an obstetric outpatient clinic for a routine prenatal check-up. Her blood pressure is measured at 145/90 mmHg using an automated office cuff. Concerned about possible gestational hypertension, her physician schedules further monitoring. However, the elevated reading was due to anxiety triggered by the clinical setting—a classic case of white-coat hypertension. Over the following days, she measures her blood pressure at home using a validated device under calm conditions, with average readings consistently around 118/76 mmHg. If a researcher reuses only the office measurement from the hospital EHR without context—such as gestational age, clinical setting, or subsequent home monitoring—the patient may be wrongly classified as hypertensive. This could affect studies on hypertensive disorders of pregnancy, overestimate prevalence, or bias models predicting maternal or fetal outcomes. If the researcher is interested in taking into account hypertension as a covariate in a risk model for infertility, pregnant women may need to be excluded all together, and the researcher needs to look for contextual clues to establish pregnancy.

A 67-year-old man visits the emergency department for chest pain. His first blood pressure is measured at 160/100 mmHg using an automated cuff. After diagnostic evaluation, the patient turns out to have a panic attack. Follow-up measurements show blood pressure values within the normal range. The elevated reading was due to the physiological stress response of the patient. If a researcher assigns the label “hypertension” to this patient, as they ever crossed the diagnostic threshold, this patient may wrongly be classified as having hypertension. This could affect case-control studies of hypertension, and studies where hypertension was included as a risk factor, for example, for cardiovascular disease.

A 74-year-old woman is admitted to the ICU with septic shock. During the first 24 h, she receives vasopressors and aggressive fluid resuscitation. Her recorded blood pressure fluctuates widely, with one of the lowest values being 78/46 mmHg. As her condition stabilizes over the next several days, her blood pressure normalizes to around 125/75 mmHg without support. If a researcher wants to assess a personalized normal value for this patient and would simply use the first available value, this woman would be labeled as “severely hypotensive”. However, this label reflects her acute critical illness, not her typical baseline. One could question the decision of the researcher to include ICU data to establish a baseline blood pressure, as including measurements in critical situations is prone for misclassification and bias.

A 52-year-old man with obesity visits hypertension outpatient clinic, where his blood pressure is first measured using a regular cuff that is too small for his arm circumference, resulting in an elevated reading of 155/95 mmHg. At a follow-up visit, a correctly sized cuff is used, and his blood pressure measures 135/85 mmHg, better reflecting his true level. This example illustrates a common issue: the selection of appropriate cuff size for blood pressure measurement is frequently neglected. A randomized crossover trial found that using a single cuff size regardless of arm circumference produces strikingly inaccurate readings, causing systematic over- or under-estimation (29). When researchers mine EHRs for blood pressure values, the cuff size is almost never recorded in structured form. That means that in a large dataset, there may be a mix of readings taken with the correct cuffs, too-small cuffs, and too-large cuffs, but it is impossible to differentiate them. Because cuff size selection depends on patient characteristics like obesity or body build, this may lead to differential misclassification: overestimating hypertension in heavier patients and underestimating it in smaller-framed individuals, potentially distorting prevalence estimates and associations (e.g., inflated BP-BMI correlation simply due to measurement artefact).

While these examples illustrate only a fraction of the complexities involved and may be self-evident to some, they serve as an important gateway for a growing body of non-medical researchers in healthcare, to consider the critical role of contextual factors in interpreting data from EHRs.

## 5 Discussion

Reusing EHR data holds great potential to advance research and improve patient care. However, as exemplified here using blood pressure measurements, quantitative EHR data may not be as straightforward as they may appear. Selecting data that truly fit the research question—data that are “fit-for-purpose”—is essential. By acknowledging the complexities inherent in healthcare data and making informed data selection decisions, researchers can move closer to unlocking the full potential of EHR data, generating meaningful findings that ultimately improve patient care.

The ideal dataset thus contains rich contextual data to be able to select data that are fit for the intended purpose. However, data selection is only the first step of preprocessing when reusing EHR data. The importance of contextual clues is not limited to the data selection step. In downstream preprocessing steps, for example during feature extraction and handling missing data (e.g., imputation strategies), these contextual data are equally important to assess the preferred strategy.

Furthermore, interpreting and discovering contextual clues may be difficult for scientists with limited knowledge of pathophysiological processes. Therefore, preprocessing of EHR data needs interdisciplinary collaboration. This interdisciplinary team often starts with clinicians who generate the data, and scientists who analyze it. For some research questions, including patients may be beneficial. Yet when reusing EHR data, the interdisciplinary team should be aware of the importance of interpreting and discovering contextual clues, for which they lack expertise, and inclusion of data managers, business intelligence officers or data platform engineers that curate and extract data may be of fundamental additional benefit. The interdisciplinary team thus ensures accurate interpretation and alignment of data and research question, including revealing gaps in contextual information and coming up with appropriate solutions.

Researchers should always strive to include the optimal data in their models, as the saying goes “garbage in = garbage out”. Future studies could aim to quantify the impact of different data selection methods on model weights, model performance, and simulated clinical decisions to further study this principle in different use cases.

## Data Availability

The original contributions presented in the study are included in the article/Supplementary Material, further inquiries can be directed to the corresponding author.
