# Supplementary material for: Beyond the numbers: the importance of contextual data when reusing blood pressure data from electronic health records
Source: Front Digit Health. 2025 Sep 3;7:1664213. doi: 10.3389/fdgth.2025.1664213 (PMC12440906; doi:10.3389/fdgth.2025.1664213)
Supplement: Supplementary file 1 [file Table1.docx]

**Table S1.** Measurement considerations (Partially based on Table 1 from Tolonen et al. (1))

| **Nr.** | **Factor** | **Method** | **Reason** | **Effect on SBP and DBP** | | **Occurrence** |
| --- | --- | --- | --- | --- | --- | --- |
| 1 | Cuff too small | AU;OSC | Artefact | ↑ 30 mmHg (2) | ↑ 30 mmHg (2) | The most frequent error in measuring office blood pressure is "miscuffing," which refers to using a cuff that is the wrong size, with undercuffing accounting for 84% of miscuffings (3). |
| 2 | Cuff too large | AU;OSC | Artefact | ↓ 10-30 mmHg (2) | ↓ 10-30 mmHg (2) |  |
| 3 | Calibration error | AU;OSC | Artefact | ~0-5 mmHg (4,5) | ~0-5 mmHg (4,5) |  |
| 4 | Calibration error | INV | Artefact | Highly variable | | Invasive line must be calibrated with the monitor. When they are calibrated incorrectly the artefact can be substantial in any direction. |
| 5 | Sensor level | INV | Artefact | Highly variable | | After calibration the sensor must be level with site you want to represent the blood pressure (typically the level of the middle of the heart). Above or below that level decreases or increases the blood pressure. |
| 6 | Underdamping / overdamping | INV | Artefact | Highly variable effect, either SBP ↑ & DBP ↓ or SBP ↓ & DBP ↑ | | The pressure wave has to be transmitted to the sensor to tubing. The sensor expects a particular amount dampening of the pressure wave. Underdampening can greatly exaggerate the pulsatility of blood pressure (including resonance phenomena), while overdamping mainly decreases the pulsatility. |
| 7 | Occlusion | INV | Artefact | No measurement or erroneous | | The occlusion can be in the catheter, but also at the orifice of the catheter. |
| 8 | External pressure | ALL | Artefact | Downward, highly variable | | External pressure to either the artery or to the cuff can greatly influence the measurement. In auscultatory measurements a common cause is the stethoscope, whereas in oscillatory measurements something of someone leaning against the cuff may be the cause (e.g. a surgeon operating on that side). |
| 9 | Auscultatory gap | AU | Artefact | SBP ↓ |  | In hypertensive patients a sudden temporary disappearance of the Korotkoff sounds may occur and cause underestimation of the systolic blood pressure. Palpation |
| 10 | Too rapid cuff deflation | AU | Artefact | SBP ↓ and/or DBP ↓ | | Deflating the cuff too quickly results in later detection of the Korotkoff sounds. |
| 11 | Arrythmias | AU;OSC | Artefact | Highly variable, typically downward |  | This is related to a patient condition, typical atrial fibrillation. Due to the arrythmia the oscillometric device or the professional is not capable of measuring the blood pressure correctly. It is a technical limitation of relying on a (fairly) regular rhythm. |
| 12 | Manual data entry error | ALL | Data entry | Highly variable | Highly variable |  |
| 13 | Supine vs. seated | ALL | Situational / procedural | Several older studies found a higher blood pressure in supine position (6–8) while newer studies found a higher blood pressure in the sitting position (9–11). | |  |
| 14 | Unsupported back/feet | ALL | Situational / procedural | ↑ 5-15 mmHg (12) | ↑ 6 mmHg (12) |  |
| 15 | Arm below heart level | ALL | Situational / procedural | ↑ 10 mmHg (2,13,14) | ↑ 10 mmHg (2,13,14) |  |
| 16 | Arm above heart level | ALL | Situational / procedural | ↑ | ↑ |  |
| 17 | Legs crossed | ALL | Situational / procedural | ↑ 5-8 mmHg (15) | ↑ 3-5 mmHg (15) |  |
| 18 | Talking during measurement | ALL | Situational / procedural | ↑ 17 mmHg (12,16) | ↑ 13 mmHg (12,16) |  |
| 19 | Left or right arm | ALL | Situational / procedural | ↓ 1–3 mmHg (17) | ↑ 1 mmHg (17) | Approximately 11.2% among hypertensive patients and 3.2 among the general population showed persistent clinically significant (SBP or DBP difference of more than 10 mmHg) interarm differences (18). A large difference in blood pressure between the arms could indicate serious pathology (see patient factors). |
| 20 | Essential hypertension | ALL | Pathological | ↑ | ↑ | A multifactorial disease of which vascular wall stiffness due to arteriosclerosis is the traditional cause. |
| 21 | Smoking | ALL | Pathological | ↑ 10 mmHg (19) | ↑ 8 mmHg (19) | Nicotine stimulates the release of adrenaline (epinephrine) in the body, which causes blood vessels to constrict. This constriction increases vascular resistance, resulting in higher blood pressure. |
| 22 | Interarm differences | ALL | Pathological | Dependent on pathology | | A large difference in blood pressure between the arms could indicate peripheral artery disease, aortic dissection, congenital aorta pathology, inflammatory artery disease, thoracic outlet syndrome. These conditions cause blood pressure to be higher in one arm than the other due to disrupted circulation (3). |
| 23 | Medication / drugs | ALL | Pathological | Highly variable | Highly variable | Various prescription and consumer drugs influence blood pressure. An extreme example of this is anesthesia, where the anesthetic drugs have a pronounced influence on blood pressure. |
| 24 | Other causes of hypertension | ALL | Pathological | Highly variable | Highly variable | Several causes which are not further specified: renal disease, endocrine disease, neurological disease, obstructive sleep apnea, vascular (e.g. anatomical, vasculitis) |
| 25 | Heart failure | ALL | Pathological | ↓ | ↓ | Various types of heart failure lower blood pressure. |
| 26 | Sepsis | ALL | Pathological | ↓ | ↓ | Systemic infection causing sepsis. |
| 27 | Trauma / bleeding | ALL | Pathological | ↓ | ↓ | Loss of blood depletes blood volume and lowers blood pressure. This may be caused by accidents, violence or medical procedures like surgery. |
| 28 | Obstructive | ALL | Pathological | ↓ | ↓ | Pulmonary embolism, heart tamponade, tension pneumothorax. |
| 29 | Liver disease | ALL | Pathological | Early stage ↑ Late stage ↓ | Early stage ↑ Late stage ↓ | Early-stage liver disease may cause high blood pressure, whereas liver cirrhosis and liver failure typically result in hypotension |
| 30 | Orthostatic hypotension | ALL | Pathological | Downward during sudden posture change |  |  |
| 31 | Other causes of hypotension | ALL | Pathological | ↓ | ↓ | Allergic reaction (anaphylaxis), endocrine, metabolic, neurological including neurogenic shock. |
| 32 | White-coat hypertension | ALL | Stress / Physiological | ↑ | ↑ | This was found in 15% among the general population and 30-40% among those with hypertension (20). |
| 33 | Masked hypertension | ALL | Stress / Physiological | ↓ | ↓ | Research estimates that 15-30% of adults with a non-elevated office blood pressure suffer from masked hypertension (21). |
| 34 | Pain | ALL | Stress / Physiological | ↑↑ | ↑ |  |
| 35 | Sports / Physical Exertion | ALL | Stress / Physiological | ↑↑ | ↑ | Recent physical activity or failure to rest for several minutes before measurement can lead to elevations in blood pressure. |
| 36 | Emotional stress and fear | ALL | Stress / Physiological | ↑↑ | ↑ |  |
| 37 | Pregnancy | ALL | Stress / Physiological | Variable | Variable | Both hypotension and hypertension may occur due to pregnancy. |
| 38 | Circadian variation | ALL | Stress / Physiological | Variable | Variable | Blood pressure is dependent on sleep/wake cycles. |
| 39 | Heavy meal before measurement | ALL | Stress / Physiological | ↓ 20 mmHg (19) | ↓ 20 mmHg (19) | Some people experience a sustained drop in blood pressure after eating. In these cases, as blood is redirected to the digestive tract, the heart rate does not increase sufficiently, and the blood vessels do not constrict enough to maintain normal blood pressure levels throughout the body. This is called postprandial hypotension and affects approximately 40.5% of older adults (22). |
| 40 | Full bladder | ALL | Stress / Physiological | ↑ 10-15 mmHg (12,19) | ↑ 10 mmHg (12,19) | A classical cause of stress in patients. |
| 41 | Relying on a single reading | AU;OSC | Situational / Procedural | Variable, typically upward | Variable, typically upward | Relying on only one blood pressure measurement instead of averaging multiple readings can misrepresent true blood pressure (23,24). |

AU = Auscultatory blood pressure measurement; OSC = Oscillometric blood pressure measurement; INV = Invasive blood pressure measurement; All of the previous methods included

1. Tolonen H, Koponen P, Naska A, Männistö S, Broda G, Palosaari T, et al. Challenges in standardization of blood pressure measurement at the population level. BMC Med Res Methodol. 2015 Apr 10;15:33. doi: 10.1186/s12874-015-0020-3
2. O’Brien E, Asmar R, Beilin L, Imai Y, Mallion JM, Mancia G, et al. European Society of Hypertension recommendations for conventional, ambulatory and home blood pressure measurement. J Hypertens. 2003 May;21(5):821–48. doi: 10.1097/00004872-200305000-00001
3. Muntner P, Shimbo D, Carey RM, Charleston JB, Gaillard T, Misra S, et al. Measurement of blood pressure in humans: a scientific statement from the American Heart Association. Hypertension. 2019 May;73(5):e35–66. doi: 10.1161/HYP.0000000000000087
4. Coleman AJ, Steel SD, Ashworth M, Vowler SL, Shennan A. Accuracy of the pressure scale of sphygmomanometers in clinical use within primary care. Blood Press Monit. 2005 Aug;10(4):181-8. doi: 10.1097/01.mbp.0000168398.87167.c2
5. de Greeff A, Lorde I, Wilton A, Seed P, Coleman AJ, Shennan AH. Calibration accuracy of hospital-based non-invasive blood pressure measuring devices. J Hum Hypertens. 2010 Jan;24(1):58-63. doi: 10.1038/jhh.2009.29
6. Netea RT, Lenders JWM, Smits P, Thien T. Both body and arm position significantly influence blood pressure measurement. J Hum Hypertens. 2003 Jul;17(7):459–62. doi: 10.1038/sj.jhh.1001573
7. Eşer I, Khorshid L, Yapucu Güneş Ü, Demir Y. The effect of different body positions on blood pressure. J Clin Nurs. 2007 Jan;16(1):137–40. doi: 10.1111/j.1365-2702.2005.01494.x
8. Wei TM, Lu LC, Ye XL, Li S, Wang L. Impact of postures on blood pressure in healthy subjects. Acta Clin Belg. 2008 Nov-Dec;63(6):376–80. doi: 10.1179/acb.2008.078
9. Lacruz ME, Kluttig A, Kuss O, Tiller D, Medenwald D, Nuding S, et al. Short-term blood pressure variability - variation between arm side, body position and successive measurements: a population-based cohort study. BMC Cardiovasc Disord. 2017 Jan 18;17(1):31. doi: 10.1186/s12872-017-0468-7
10. Privšek E, Hellgren M, Råstam L, Lindblad U, Daka B. Epidemiological and clinical implications of blood pressure measured in seated versus supine position. Medicine (Baltimore). 2018 Aug;97(31):e11603. doi: 10.1097/MD.0000000000011603
11. Krzesiñski P, Stañczyk A, Gielerak G, Piotrowicz K, Banak M, Wójcik A. The diagnostic value of supine blood pressure in hypertension. Arch Med Sci. 2016 Apr 1;12(2):310-8. doi: 10.5114/aoms.2016.59256
12. Handler J. The importance of accurate blood pressure measurement. Perm J. 2009 Summer;13(3):51–4. doi: 10.7812/TPP/09-054
13. Netea RT, Lenders JWM, Smits P, Thien T. Arm position is important for blood pressure measurement. J Hum Hypertens. 1999 Feb;13(2):105–9. doi: 10.1038/sj.jhh.1000720
14. Netea RT, Smits P, Lenders JWM, Thien T. Does it matter whether blood pressure measurements are taken with subjects sitting or supine? J Hypertens. 1998 Mar;16(3):263-8. doi: 10.1097/00004872-199816030-00002
15. Keele-Smith R, Price-Daniel C. Effects of crossing legs on blood pressure measurement. Clin Nurs Res. 2001 May;10(2):202-213. doi: 10.1177/C10N2R8
16. Schulze MB, Kroke A, Saracci R, Boeing H. The effect of differences in measurement procedure on the comparability of blood pressure estimates in multi-centre studies. Blood Press Monit. 2002 Apr;7(2):95–104. doi: 10.1097/00126097-200204000-00002
17. Gould BA, Hornung RS, Kleso HA, Altman DG, Raftery EB. Is the blood pressure the same in both arms? Clin Cardiol. 1985 Aug;8(8):423–6. doi: 10.1002/clc.4960080803.
18. Clark CE, Taylor RS, Campbell JL, Shore AC. Prevalence of systolic inter-arm differences in blood pressure for different primary care populations: systematic review and meta-analysis. Br J Gen Pract. 2016 Nov;66(652):e838–47. doi: 10.3399/bjgp16X687553
19. Campbell NR, McKay DW, Chockalingam A, Fodor JG. Errors in assessment of blood pressure: patient factors. Can J Public Health. 1994 Sep-Oct;85 Suppl 2:S12-7.
20. Mancia G, Facchetti R, Bombelli M, Cuspidi C, Grassi G. White-coat hypertension: pathophysiological and clinical aspects: excellence award for hypertension research 2020. Hypertension. 2021 Dec;78(6):1677–88. doi: 10.1161/HYPERTENSIONAHA.121.16489
21. Peacock J, Diaz KM, Viera AJ, Schwartz JE, Shimbo D. Unmasking masked hypertension: prevalence, clinical implications, diagnosis, correlates and future directions. J Hum Hypertens. 2014 Sep;28(9):521–8. doi: 10.1038/jhh.2014.9.
22. Huang L, Li S, Xie X, Huang X, Xiao LD, Zou Y, et al. Prevalence of postprandial hypotension in older adults: a systematic review and meta-analysis. Age Ageing. 2024 Feb 1;53(2):afae022. doi: 10.1093/ageing/afae022
23. Hong G, Hansen B, Gulati M. The delusion of measuring blood pressure. British Journal of Cardiology. 2023 Aug 9;30(3):23. doi: 10.5837/bjc.2023.023
24. Elias MF, Goodell AL. Human Errors in Automated Office Blood Pressure Measurement: Still Room for Improvement. Hypertension. 2021. Jan;77(1):6-15. doi: 10.1161/HYPERTENSIONAHA.120.16164
